# Informing evidence-based policies for ageing and health in Ghana

**DOI:** 10.2471/BLT.14.136242

**Published:** 2014-10-27

**Authors:** Islene Araujo de Carvalho, Julie Byles, Charles Aquah, George Amofah, Richard Biritwum, Ulysses Panisset, James Goodwin, John Beard

**Affiliations:** a World Health Organization, avenue Appia 20, 1211 Geneva 27, Switzerland.; b Research Centre for Gender, Health & Ageing, University of Newcastle, England.; c Ghana Health Service, Accra, Ghana.; d Accra, Ghana.; e University of Ghana Medical School, Accra, Ghana.; f Age UK, London, England.

## Abstract

**Problem:**

Ghana’s population is ageing. In 2011, the Government of Ghana requested technical support from the World Health Organization (WHO) to help revise national policies on ageing and health.

**Approach:**

We applied WHO’s knowledge translation framework on ageing and health to assist evidence based policy-making in Ghana. First, we defined priority problems and health system responses by performing a country assessment of epidemiologic data, policy review, site visits and interviews of key informants. Second, we gathered evidence on effective health systems interventions in low- middle- and high-income countries. Third, key stakeholders were engaged in a policy dialogue. Fourth, policy briefs were developed and presented to the Ghana Health Services.

**Local setting:**

Ghana has a well-structured health system that can adapt to meet the health care needs of older people.

**Relevant changes:**

Six problems were selected as priorities, however after the policy dialogue, only five were agreed as priorities by the stakeholders. The key stakeholders drafted evidence-based policy recommendations that were used to develop policy briefs. The briefs were presented to the Ghana Health Service in 2014.

**Lessons learnt:**

The framework can be used to build local capacity on evidence-informed policy-making. However, knowledge translation tools need further development to be used in low-income countries and in the field of ageing. The terms and language of the tools need to be adapted to local contexts. Evidence for health system interventions on ageing populations is very limited, particularly for low- and middle-income settings.

## Résumé

**Problème:**

La population du Ghana est vieillissante. En 2011, le gouvernement du Ghana a sollicité l'assistance technique de l'Organisation mondiale de la Santé (OMS) pour l'aider à réviser ses politiques nationales relatives au vieillissement et à la santé.

**Approche:**

Nous avons appliqué le cadre d'application des connaissances de l'OMS sur le vieillissement et la santé pour aider à élaborer des politiques fondées sur les données au Ghana. Premièrement, nous avons défini les problèmes prioritaires et les réponses du système de santé en réalisant une évaluation nationale des données épidémiologiques, une révision des politiques, des visites sur site et des entrevues menées auprès des informateurs clés. Deuxièmement, nous avons recueilli les données sur les interventions efficaces des systèmes de santé dans les pays à revenu faible, intermédiaire et élevé. Troisièmement, les principales parties prenantes ont été engagées dans un dialogue politique. Quatrièmement, des documents politiques ont été élaborés et présentés aux Services de Santé du Ghana.

**Environnement local:**

Le Ghana possède un système de santé bien structuré, qui peut s'adapter pour répondre aux besoins des personnes âgées en matière de soins de santé.

**Changements significatifs:**

Six problèmes ont été sélectionnés comme prioritaires. Cependant, après le dialogue politique, seuls cinq d'entre eux ont été acceptés comme prioritaires par les parties prenantes. Les principales parties prenantes ont rédigé des recommandations pour les politiques fondées sur les données qui ont été utilisées pour élaborer les documents politiques. Les documents ont été présentés aux Services de Santé du Ghana en 2014.

**Leçons tirées:**

Le cadre peut être utilisé pour renforcer les capacités locales en matière d'élaboration des politiques fondées sur les données. Cependant, les outils d'application des connaissances ont besoin d'être davantage développés pour être utilisés dans les pays à revenu faible et dans le domaine du vieillissement. Les termes et la langue des outils doivent être adaptés aux contextes locaux. Les données des interventions du système de santé sur les populations vieillissantes sont très limitées, en particulier pour les pays à revenu faible et intermédiaire.

## Resumen

**Situación:**

La población de Ghana está envejeciendo. En 2011, el Gobierno de Ghana solicitó la asistencia técnica de la Organización Mundial de la Salud (OMS) para ayudar a revisar las políticas nacionales sobre el envejecimiento y la salud.

**Enfoque:**

Aplicamos el marco de traducción de conocimientos de la OMS sobre el envejecimiento y la salud para apoyar la formulación de políticas basadas en datos empíricos en Ghana. En primer lugar, definimos los problemas prioritarios y las respuestas del sistema de salud mediante la realización de una evaluación nacional de los datos epidemiológicos, una revisión de la política, visitas de campo y entrevistas a informantes clave. En segundo lugar, recopilamos datos empíricos sobre las intervenciones de los sistemas de salud eficaces en países de ingresos bajos, medios y altos. En tercer lugar, las principales partes interesadas entablaron un diálogo sobre políticas y, en cuarto lugar, se desarrollaron y presentaron informes de políticas a los Servicios de Salud de Ghana.

**Marco regional:**

Ghana tiene un sistema de salud bien organizado, capaz de adaptarse para atender las necesidades de asistencia sanitaria de las personas mayores.

**Cambios importantes:**

Se seleccionaron seis problemas como prioridades, sin embargo después del diálogo sobre políticas, las partes interesadas acordaron solo cinco de ellos como prioritarios. Las principales partes interesadas redactaron recomendaciones políticas basadas en datos empíricos que se usaron para desarrollar informes sobre políticas. Los escritos se presentaron al Servicio de Salud de Ghana en 2014.

**Lecciones aprendidas:**

El marco puede utilizarse para potenciar las capacidades locales en la formulación de políticas basadas en datos empíricos. Sin embargo, las herramientas de traducción de conocimientos necesitan un mayor desarrollo para emplearse en los países de ingresos bajos y en el ámbito del envejecimiento. Los términos y el lenguaje de las herramientas deben adaptarse a los contextos locales. Los datos empíricos acerca de las intervenciones del sistema de salud sobre el envejecimiento de la población son muy limitados, sobre todo para los contextos de ingresos bajos y medios.

## ملخص

**المشكلة:**

يشيخ سكان غانا. وفي عام 2011، طلبت حكومة غانا الدعم التقني من منظمة الصحة العالمية للمساعدة في تنقيح السياسات الوطنية بشأن الشيخوخة والصحة.

**الأسلوب:**

قمنا بتطبيق إطار ترجمة معارف منظمة الصحة العالمية بشأن الشيخوخة والصحة للمساعدة في وضع السياسات المسندة إلى البيّنات في غانا. أولاً، قمنا بتحديد المشكلات ذات الأولوية وأوجه استجابة النظام الصحي عن طريق إجراء تقييم قطري للبيانات الوبائية واستعراض السياسة والزيارات الميدانية والمقابلات التي أجريت مع مبلغي المعلومات الرئيسيين. ثانياً، قمنا بتجميع البيّنات بشأن تدخلات النظم الصحية الفعالة في البلدان المنخفضة الدخل والبلدان المتوسطة الدخل والبلدان المرتفعة الدخل. ثالثاً، تم إشراك أصحاب المصلحة الرئيسيين في حوار حول السياسات. رابعاً، تم وضع تقارير موجزة عن السياسات وتقديمها إلى الخدمات الصحية في غانا.

**المواقع المحلية:**

يوجد لدى غانا نظام صحي منظم بشكل جيد يمكنه التكيف لتلبية احتياجات الرعاية الصحية للمسنين.

**التغيّرات ذات الصلة:**

تم تحديد ست مشكلات كأولويات، غير أنه بعد الحوار الذي أجري حول السياسات، لم يتم الاتفاق من جانب أصحاب المصلحة سوى على خمس منها كأولويات. وقام أصحاب المصلحة الرئيسيين بوضع مسودة التوصيات السياسية المسندة إلى البيّنات التي استخدمت لوضع التقارير الموجزة عن السياسات. وتم تقديم التقارير الموجزة إلى الخدمة الصحية في غانا في عام 2014.

**الدروس المستفادة:**

يمكن استخدام الإطار لبناء القدرة المحلية بشأن وضع السياسات المستنيرة بالبيّنات. ومع ذلك، يجب مواصلة تطوير أدوات ترجمة المعارف لاستخدامها في البلدان المنخفضة الدخل وفي مجال الشيخوخة. ويجب تكييف مصطلحات الأدوات ولغتها حسب السياقات المحلية. وتتسم البيّنات الخاصة بتدخلات النظم الصحية بشأن الفئات السكانية المسنة بالمحدودية الشديدة، لا سيما بخصوص البيئات المنخفضة الدخل والبيئات المتوسطة الدخل.

## 摘要

**问题:**

加纳人口正在老龄化。2011年，加纳政府请求世界卫生组织（WHO）提供技术支持以帮助修订有关老龄化和卫生的国家政策。

**方法:**

我们应用WHO有关老龄化与卫生的知识转化框架来帮助加纳的询证决策。首先，我们通过执行国家流行病学数据评估、政策评估、现场走访和面访关键信息提供者来定义优先级问题和卫生系统的回应。其次，我们收集中低收入和高收入国家有关有效卫生系统干预措施的证据。第三，召集关键利益相关者参与政策对话。第四，编制政策简报并呈递给加纳卫生总局。

**当地状况:**

加纳卫生系统结构完善，通过调适能够满足老年人的卫生保健需求。

**相关变化:**

入选的优先问题有六个，然而在政策对话后，利益相关者只同意其中五个为优先问题。关键的利益相关者起草用于编制政策简报的询证政策建议。简报于 2014年呈递给加纳卫生总局。

**经验教训:**

框架可用于构建知证的地方政策制定能力。然而，知识转化工具需要进一步开发，以便在低收入国家和老龄化的领域中使用。工具的术语和语言需要与当地的环境相适应。卫生系统人口老龄化干预措施的证据非常有限，在中低收入背景中尤其如此。

## Резюме

**Проблема:**

Население Ганы стареет. В 2011 году правительство Ганы запросило техническую поддержку у Всемирной организации здравоохранения (ВОЗ) для помощи в пересмотре национальной политики в области старения и здравоохранения.

**Подход:**

Была использована модель преобразования знаний ВОЗ по проблемам старения и здоровья для помощи в разработке доказательно-обоснованной политики в Гане. Во‑первых, были определены приоритетные проблемы и реакция системы здравоохранения путем выполнения страновой оценки эпидемиологических данных, обзора политики, посещения мест и опросов ключевых источников информации. Во‑вторых, были собраны свидетельства эффективных мероприятий в системах здравоохранения в странах с низким, средним и высоким уровнями доходов. В-третьих, в диалог по разработке политики были вовлечены основные заинтересованные стороны. В‑четвертых, были разработаны концепции политики, которые затем были представлены Службе здравоохранения Ганы.

**Местные условия:**

Гана располагает хорошо структурированной системой здравоохранения, которая может адаптироваться для удовлетворения потребностей пожилых людей в медицинской помощи.

**Осуществленные перемены:**

В качестве приоритетных были выбраны шесть проблем, однако после диалога по вопросам разработки политики только пять из них были согласованы в качестве приоритетных заинтересованными сторонами. Основные заинтересованные стороны составили рекомендации по доказательно-обоснованной политике, которые были использованы для разработки концепций политики. Эти концепции были представлены Службе здравоохранения Ганы в 2014 году.

**Выводы:**

Эта модель может использоваться для создания местного потенциала для разработки фактологически-обоснованной политики. Однако, необходимо продолжить разработку инструментов преобразования знаний, которые будут использоваться в странах с низким уровнем дохода и в области старения. Термины и язык инструментов должны быть адаптированы к местным условиям. Фактические данные для проведения мероприятий, касающихся старения населения, в системах здравоохранения весьма ограничены, особенно в странах с низким и средним уровнями доходов.

## Introduction

Population ageing presents challenges and opportunities and requires action on the part of national policy-makers.^1^ However, in low- and middle-income countries, which often have high rates of maternal mortality and infectious diseases, effective responses to the health needs of older people need to be prioritized. In addition, evidence on health systems interventions for the ageing population and evidence-based responses are often not available or applied in these countries.^2^^,^^3^

To bridge the gap between evidence and policy in the field of ageing, the World Health Organization (WHO) with the support of Age UK, produced a knowledge translation framework on ageing and health in 2012.^3^ This framework is based on the SUPPORT tools for evidence-informed health policy-making^4^ and the EVIPNet methods designed to support the development of policies based on existing research.^5^ In 2012, the Government of Ghana requested technical support to help revise and improve its existing policy and implementation plan on ageing and health. This provided an opportunity to trial the framework in a middle-income country. This paper describes the process used and the main lessons learnt.

## Approach

We defined priority health problems by assessing the needs of older people and the national health system and policy context. We did an initial informal assessment of the political environment and found that the use of knowledge translation in the country was favourable with a national ageing policy released in July 2010^6^ and an implementation plan in place. There was also good local epidemiological data on older people’s needs from the *Study on global ageing and adult health*, which includes a nationally-representative sample of 3923 Ghanaians older than 50 years.^7^ Ghana has a well-structured health system that could be extended to meet the health care needs of older people, although expertise in ageing is scarce. In 2010, 6.7% of the population in Ghana were older than 60 years (6.0% men; 7.3% women) and the proportion of older people is increasing. The country is undergoing an epidemiological transition with the emergence of noncommunicable diseases as the major cause of disease burden in this age group.

Using epidemiologic evidence, review of policy documents, site visits and interviews with key informants, we first drafted a country assessment report that identified priority problems. Second, we synthetized evidence on effective health system interventions for each identified problem. Information was obtained from the WHO package of essential noncommunicable diseases interventions for primary health care in low-resource settings^8^ and from the McMaster Health Forum’s health systems evidence database.^9^ Time and resource constraints limited our searches to only one global database. The McMaster database is the most comprehensive,^9^ free access source to evidence on how to strengthen health systems. It contains AMSTAR (A Measurement Tool to Assess Systematic Reviews)-graded systematic reviews of research about ageing interventions in low- middle- and high-income countries.

Third, the Ghana Health Service, with WHO support, organized a three-day policy dialogue to discuss the identified problems. This meeting involved representatives from key ministries, the Ghana Health Service, teaching hospitals, professional bodies, HelpAge Ghana and WHO. The dialogue was structured around clarifying the problems and framing policy options. The meeting began with an interactive plenary session on the global and African status on ageing and health as well as key findings of the assessment report and progress made during the implementation action plan of the Ghana National Ageing Policy. Groups were then formed for each problem. Participants signed up for one of the groups based on their interest and expertise. Afterwards we reviewed the composition of the groups to maintain homogeneity in numbers and the profiles of participants. The group identified the scope and underlying causes of each priority problem and reviewed which of our synthesized evidence on health interventions could be implemented in Ghana. Basic concepts of evidence-based policy-making were covered in didactic presentations by WHO staff.

Fourth, together with a small group of experts and policy-makers for Ghana, we developed policy briefs for each problem, with recommended actions for the Ministry of Health and the Ghana Health Service. This project cost approximately 100 000 United States dollars.

## Results

Using the country assessment report, we identified six priority problems, based on prevalence, impact on health, amenability to change within the country context and alignment with the 2010 Ghana National Ageing Policy: (i) undiagnosed and untreated hypertension; (ii) high prevalence of respiratory problems; (iii) limitation of physical function affecting social participation and quality of life; (iv) poor utilization of health-care services by older people; (v) inadequate preparedness of the health workforce to care for older people; and (vi) obesity.

Participants in the policy dialogue replaced obesity with visual and hearing impairment. While the latter did not emerge as a priority from the available epidemiologic data, these conditions are common elsewhere. Studies from Ghana suggest that approximately 8.0% of outpatients reported hearing issues,^10^ and experts believe that the proportion of people with visual impairment is high.

Similarly, while dementia was not identified as a priority problem, participants felt it should be specified within the limitation of physical function priority. This is because there is a low awareness of the problem and there are limited services for people with dementia.

When participants were divided into working groups, there was no interest in the respiratory diseases problem. Consequently, this problem was not addressed. The exclusion of respiratory problems from the agenda does not mean they are not a prevalent and important health concern for the people in Ghana, but that the context did not support a policy response to this problem at this time.

The groups developed a more nuanced understanding of the problems and reframed some of them. For example, the problem of undiagnosed and untreated hypertension was reframed as high prevalence and low control of hypertension, since the problem was not simply under-diagnosis, but also inadequate long-term treatment. The *Study on global ageing and adult health* showed that 57% of people older than 50 years in Ghana had hypertension on clinical examination. However, only 23% of them were aware of their condition and of these people, 4.1% were on long-term treatment.^7^ This highlights the gap between awareness and control as well as the need for better adherence to treatment.^11^^,^^12^ Participants suggested that inadequate ongoing treatment may be due to low health insurance coverage in older Ghanaians, even though insurance is free for people older than 70 years. The *Study on global ageing and adult* *health* confirmed that insurance coverage is below 50% for people older than 60 years. Other participants discussed the difficulty of conveying the concept of chronic conditions and the need for ongoing treatment within the older people’s belief systems. Understanding of chronic disease and curability varies for different population groups in Ghana, with complex relationships between knowledge, beliefs and health practices.^13^ Consequently, the problem of hypertension was reframed from limited access to screening and drug treatments, to an issue concerning older people’s understanding of the nature of chronic disease and chronic care.

The working group’s final definition of the five priority problems were (i) undiagnosed and untreated hypertension; (ii) functional impairment and social isolation; (iii) poor utilization of health-care services by older people; (iv) inadequate preparedness of the health workforce to care for older people; and (v) high level of sensory impairment (visual impairment and hearing loss) which are undetected and/or unmanaged among the elderly.

As a next step, the groups considered policy options and interventions using evidence on cost-effective health systems responses to ageing. Direct evidence for such responses in middle-income countries is limited and generalization from higher income settings may not be appropriate. Another challenge arose from how the priorities were selected, since the selection process was mainly based on epidemiologic studies and not a health systems approach. Participants therefore restructured the meeting to allow further discussion on how to build health systems that would address the selected priorities and the health system weakness. This led to a list of key interventions (Box 1) to be included in policy briefs. The policy briefs recommended relevant health systems responses.

Box 1 **Recommended interventions for ageing and health in Ghana**

- Sensitize the community to the health needs of older adults; targeting information and education efforts to the public, carers, community leaders and religious organizations.
- Integrate ageing and health in the community health workers’ programme.
- Build workforce capacity at all levels in the health system.
- Create age-friendly health facilities.
- Broaden insurance coverage by increasing the range of services and the number of people who are eligible.
- Make devices for hearing and visual impairment available to people in need.
- Create and empower support groups to assist with screening, education, management and care of older people in communities.

The policy briefs were presented at the strategic planning retreat of the Ghana Health Service in August 2013 and key policy recommendations on ageing were incorporated into its five-year operational plan.

## Discussion

This is the first use-case of WHO’s knowledge translation framework for ageing and health. Our experience shows that the framework can be useful in a middle-income setting to guide the development of evidence-informed policy. The process engaged a wide range of stakeholders and provided a systematic and transparent approach to the appraisal, evaluation and use of evidence in decision-making. It was timed to inform the operational plan of the Ghana Health Service.

However, several challenges arose in the knowledge translation process. While the policy dialogue was well-attended, engagement of key stakeholders was difficult due to competing demands on their time. Most participants were not experienced in using research findings and could not relate to the terms used in the SUPPORT tools and EVIPNet methods. We therefore revised the tools during the meeting to include more familiar language, to enable participants to stay engaged in the knowledge translation process. Finally, the lack of evidence on health systems interventions for middle-income settings resulted in the use of more anecdotal evidence to inform the policy briefs. 

In conclusion (Box 2), this project suggests that a knowledge translation approach to develop a policy on ageing and health can be useful in middle-income settings. However, the framework needs to be adapted to local settings and more health systems research in low- and middle-income countries is needed.

Box 2 **Summary of main lessons learnt**

- The framework was useful for engaging stakeholders in a middle-income country to develop evidence-informed policies on ageing.
- The terms used in the tools need to be adapted to local contexts.
- Lack of research on health systems interventions for ageing populations in middle-income countries is a significant barrier and flexible knowledge translation methods are needed for policy development in these settings.
